# A new biomarker panel of ultraconserved long non-coding RNAs for bladder cancer prognosis by a machine learning based methodology

**DOI:** 10.1186/s12859-023-05167-6

**Published:** 2023-03-06

**Authors:** Angelo Ciaramella, Emanuel Di Nardo, Daniela Terracciano, Lia Conte, Ferdinando Febbraio, Amelia Cimmino

**Affiliations:** 1 Department of Science and Technology, University of Naples “Parthenope”, Centro Direzionale, Isola C4, 80143 Naples, Italy; 2 Department of Computer Science, University of Milan, Via Celoria, 18, 20133 Milan, Italy; 3 Department of Translational Medical Science, University of Naples “Federico II”, Via Pansini 5, 80131 Naples, Italy; 4 Department of Experimental Urology, Radboud University Medical Center, Geert Grooteplein-Zuid 10, 6525 GA Nijmegen, The Netherlands; 5 Institute of Biochemistry and Cell Biology, CNR, Via Pietro Castellino 111, 80131 Naples, Italy; 6 Institute of Genetics and Biophysics, CNR, Via Pietro Castellino 111, 80131 Naples, Italy

**Keywords:** Feature relevance, Machine learning, Ultraconserved lncRNA, Bladder cancer, Cancer prognostic biomarker

## Abstract

**Background:**

Recent studies have indicated that a special class of long non-coding RNAs (lncRNAs), namely Transcribed-Ultraconservative Regions are transcribed from specific DNA regions (T-UCRs), 100$\%$ conserved in human, mouse, and rat genomes. This is noticeable, as lncRNAs are usually poorly conserved. Despite their peculiarities, T-UCRs remain very understudied in many diseases, including cancer and, yet, it is known that dysregulation of T-UCRs is associated with cancer as well as with human neurological, cardiovascular, and developmental pathologies. We have recently reported the T-UCR uc.8+ as a potential prognostic biomarker in bladder cancer.

**Results:**

The aim of this work is to develop a methodology, based on machine learning techniques, for the selection of a predictive signature panel for bladder cancer onset. To this end, we analyzed the expression profiles of T-UCRs from surgically removed normal and bladder cancer tissues, by using custom expression microarray. Bladder tissue samples from 24 bladder cancer patients (12 Low Grade and 12 High Grade), with complete clinical data, and 17 control samples from normal bladder epithelium were analysed. After the selection of preferentially expressed and statistically significant T-UCRs, we adopted an ensemble of statistical and machine learning based approaches (i.e., logistic regression, Random Forest, XGBoost and LASSO) for ranking the most important diagnostic molecules. We identified a signature panel of 13 selected T-UCRs with altered expression profiles in cancer, able to efficiently discriminate between normal and bladder cancer patient samples. Also, using this signature panel, we classified bladder cancer patients in four groups, each characterized by a different survival extent. As expected, the group including only Low Grade bladder cancer patients had greater overall survival than patients with the majority of High Grade bladder cancer. However, a specific signature of deregulated T-UCRs identifies sub-types of bladder cancer patients with different prognosis regardless of the bladder cancer Grade.

**Conclusions:**

Here we present the results for the classification of bladder cancer (Low and High Grade) patient samples and normal bladder epithelium controls by using a machine learning application. The T-UCR’s panel can be used for learning an eXplainable Artificial Intelligent model and develop a robust decision support system for bladder cancer early diagnosis providing urinary T-UCRs data of new patients. The use of this system instead of the current methodology will result in a non-invasive approach, reducing uncomfortable procedures (such as cystoscopy) for the patients. Overall, these results raise the possibility of new automatic systems, which could help the RNA-based prognosis and/or the cancer therapy in bladder cancer patients, and demonstrate the successful application of Artificial Intelligence to the definition of an independent prognostic biomarker panel.

**Supplementary Information:**

The online version contains supplementary material available at 10.1186/s12859-023-05167-6.

## Background

**Fig. 1:**
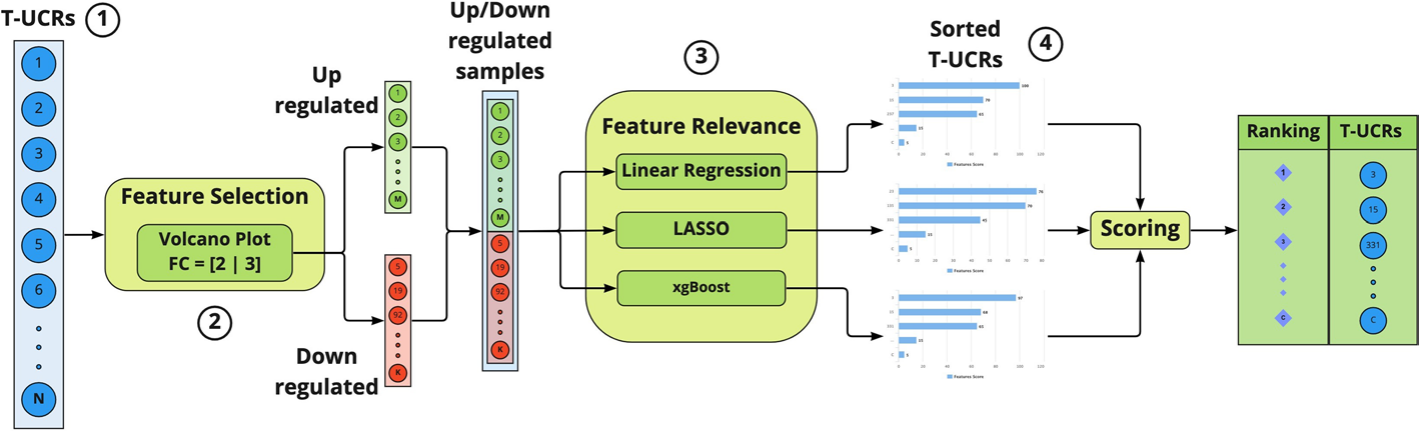
Applied methodology. The T-UCRs expressions (1) are evaluated using a volcano plot (2), most important features are retained. Features are passed (3) to an ensemble of ML models for a comparison of the features’ relevance. Scores are sorted and the estimation is provided (4)

Recently, molecular biomarkers with prognostic and predictive values in bladder cancer have been investigated because of the development of next-generation sequencing and gene expression profiling [1]. Additionally, long non-coding RNAs (lncRNAs) play a pivotal role in predicting clinical outcomes [2]. Nevertheless, a prognostic model based on the expression of lncRNAs has only marginally been studied. Thus, it is critical to determine a novel lncRNA signature to better predict the prognosis of patients with bladder cancer and to provide future targeted clinical strategies. The long non coding RNAs (lncRNAs) include the transcribed ultraconserved regions (T-UCRs) class, 481 transcripts of about 200 nt in length that are $100\%$ conserved in human, mouse and rat genomes [3]. The conservation of T-UCR is noteworthy, as lncRNAs as a class are typically relatively poorly conserved (30–$40\%$) and evolutionary conservation is often considered a marker of biological significance. This candidates the T-UCRs as more strictly regulated and change-sensitive biomarkers. However, most of these sequences are not still reported in databases, thus RNAseq datasets are not available to be used. Moreover, studies on T-UCRs are still in their infancy and much still needs to be done for a better understanding of their biological roles. To date, it is known that dysregulation of T-UCRs is associated with cancer [4] as well as with several human diseases including neurological, cardiovascular and developmental conditions [5]. Therefore, the appropriate modulation of T-UCR levels may hold promising cancer therapies and their relative expression levels may reflect the disease state [4, 6]. The hypothesis that a set of T-UCRs may represent a decisive information on the state of bladder cancer (BlCa) is based on our previous reports on the profile of all 481 T-UCRs on a large and well-characterized panel of BlCa tissues [4, 6]. Our results, obtained by the custom microarray technology (OSU-CCC 4.0, Ohio State University Comprehensive Cancer Center), show that many of these T-UCRs are likely drivers of BlCa development and progression, inspiring their potential use as BlCa biomarkers in diagnosis or prognosis [4, 6]. Indeed, the uc.8+ lncRNA was identified as the most upregulated T-UCR in BlCa, the most common malignancy of the urinary tract and one of the most prevalent cancers worldwide [7]. The relevance of uc.8+ is also suggested by the finding that it is $100\%$ preserved from human to elephant and $83\%$ from human to zebrafish. BlCa is a heterogeneous disease, involving complex mechanisms that are not easily manageable with efficacious therapies in a timely manner. Improving BlCa diagnosis and follow-up to significantly increase survival rates is one of the biggest challenges nowadays. Although the differences in molecular pathogenesis between non-invasive and invasive BlCa have been recognized, the identification of panels with validated markers, associated with clinical and pathological variables, may be the most promising approach for accurate risk stratification and clinical decision-making. Besides biomedical methodologies, different computational methods have been applied to biomarkers discovery [8–10], like Support Vector Machine [11], Random Forest [8, 12], that uses “decision trees” for categorizing the data, and to select the most important features [13], penalization techniques based on variable selection penalties [14], and Gradient Boosting [15]. Despite the large use of machine learning (ML) methodologies, to leverage data and improve performances, and the growing interest in T-UCRs in pathologies like cancer, to our knowledge no studies about T-UCRs use as biomarkers using ML methodologies are available in the literature. As recently published, we have already applied ML approaches to one T-UCR for its validation as a prognostic biomarker in BlCa [6]. In this work, we have extended the ML-based methodology to the analysis of a whole set of T-UCRs microarray data for the identification of a panel of biomarkers to be used in BlCa prognosis. The aim was to define a biomarker’s panel selecting small M-dimensional datasets of T-UCRs from the whole N-dimensional dataset of T-UCRs, where $M \ll N$, by using feature selection and feature relevance techniques (Fig. 1). In this work, we considered features selection methods focused on relevant features to be adapted to small data sets, robust to noise thus obtaining an explainable result (see Section 1 of Additional file 1 for a brief overview of the methods and their characteristics). In this respect, we used four approaches (i.e., logistic regression, LASSO, Random Forest, XGBoost) to improve, integrate and optimize the estimation of the T-UCRs panel. The resulting panel of T-UCRs was successfully applied to the classification of patients with respect to their survival, returning a grade-independent, more confident BlCa prognosis (see https://github.com/CI-SSLab/bladder_cancer_tucr_xai for this software).

### T-UCRs panel selection

The analyses performed by the ensemble models permitted to rank the T-UCRs with respect to the relevance of their altered expression in BlCa patients. In order to merge the results from the different methodologies, we assigned a score at each T-UCRs depending on the rank they obtained in each of the ensemble of feature importance models. We associated higher rank coinciding with higher score and we considered that the models of the ensemble have comparable significance. Formally, the sum of the scores obtained by a T-UCR for each methodology, was used to define a new ranking of significance for the T-UCRs which takes into account the results of all the used methodologies, meaning that we don’t operate penalties or normalization. A further cut-off was applied to the ranking generated from the estimation of the four methodologies, in order to shrink the number of significant T-UCRs (i.e., 13 and 21, respectively). This estimate allows us to define a number of selected T-UCRs with altered expression profiles in cancer as a possible signature panel able to describe the difference between NBE and LG or HG BlCa patients.

### BlCa patients survival and statistical analysis

The survival analysis of BlCa patients based on the T-UCRs differential expression signature panel was assessed using Kaplan-Meier survival analysis. Survival data, in months, of the LG and HG patients have been used. The log-rank *p* values were determined assuming a null hypothesis that there are no differences at all among the groups. Python scripts based on “lifelines” software [16] were used to plot the data and calculate the log-rank *p* value of the patients survival using Kaplan-Meier analysis.

## Experimental results

In this section we describe the results obtained for selecting the representative T-UCRs from real data.

### Feature selection

**Fig. 2:**
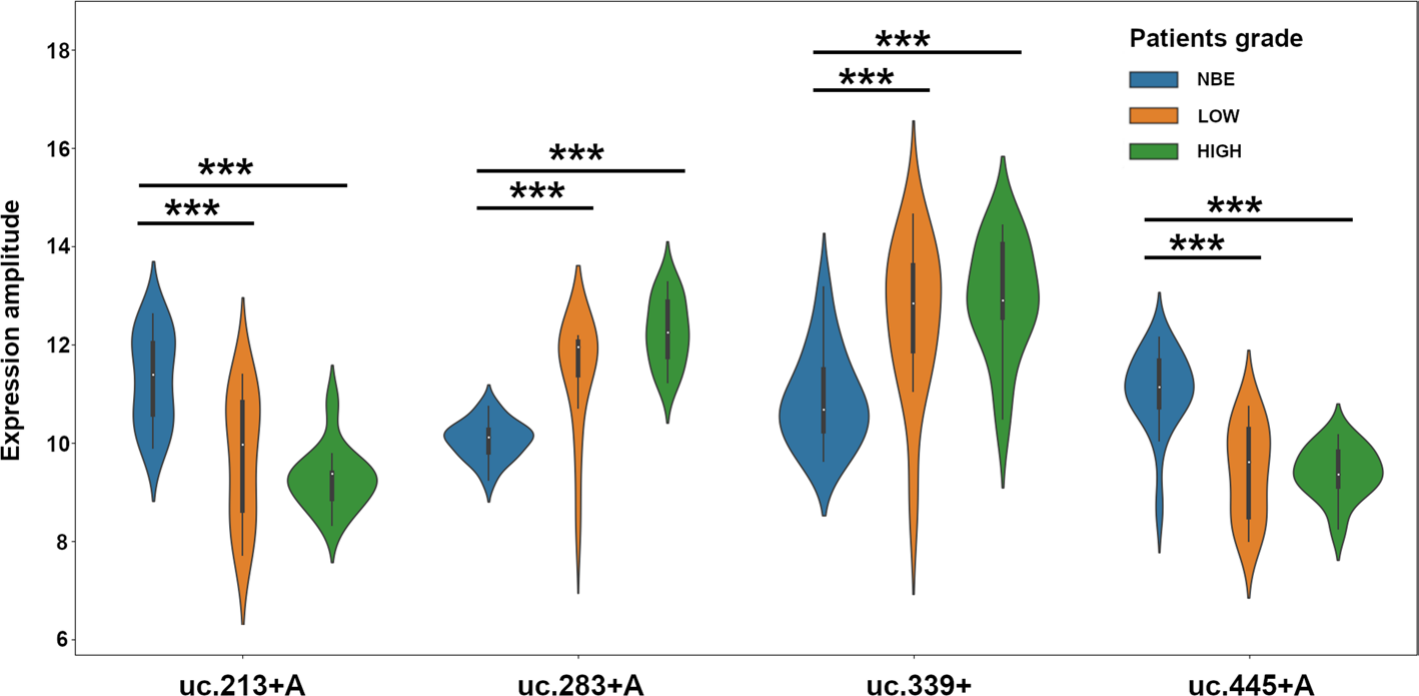
Violin plot of the differential expressions of T-UCRs 213+A, 283+A, 339+ and 445+A, in Control samples (NBE), and LOW and HIGH Grade BlCa patients. *** indicated *p* values $< 0.001$

We analyzed the Volcano plot comparing gene expression sets. We used a *p* value of 0.001 and 0.005 for each comparison and a FC threshold of 2 and 3 (where in the graph 1 and $1.58 = \log _2(FC)$, respectively). As example in Fig. 6a, b we show the Volcano plot with cut-off criteria $p\,value < 0.001$ and $\log _2(FC) > 1.58$ considering LG BlCa vs. NBE, and HG BlCa vs. NBE patients, respectively. In our experiments, we observed that by varying the *p* value, the panel of selected T-UCRs remains unchanged. By varying the FC from 2 to 3, we obtain a more robust panel with 70 and 24 T-UCRs, respectively. We also observe that gene expressions, such as uc.8+, appear always up-regulated, which may indicate a significant involvement of this T-UCR in tumor development than the other T-UCRs. Moreover, the expression of the most of the T-UCRs with significant FC values appears different depending on the control or patient samples. In fact, as shown by the violin plot in Fig. 2 of the T-UCRs 213+A, 283+A, 339+ and 445+A expression, the range of possible values change between the NBE and BlCa patients. Also, we can observe differences between samples from patients diagnosed with LG or HG BlCa, such as 283+A which increase following NBE<LG<HG or 213+A which decrease following NBE>LG>HG.

### Feature relevance and scoring

Feature relevance covers a key role in several fields, making it possible to treat models with fewer variables and to assign scores to input features for building a predictive model. This is fundamental for a better understanding of the model in terms of interpretability and eXplainability. Its application to biological data, such as cancer biomarkers, could be useful to diagnose and prognosis in many types of cancer. In order to use the differences of T-UCRs expression like a tool for improving the prognosis of the disease progression in BlCa patients, we need to rank the most significant T-UCRs identified by the volcano plot (section) using the $p\,value < 0.001$ and the two $\log _2(FC)$. Feature importance is used to improve a predictive model selecting those T-UCRs to delete (lowest scores) or those T-UCRs to keep (highest scores).

We created two lists of genes to have different degrees of pre-selection of T-UCRs and to confirm, with more robust analysis, the ranking of the most significant T-UCRs.

Applying the ensemble of feature importance methodologies described in section, we ranked the 70 and 24 T-UCRs obtained varying the FC from 2 to 3, respectively. The ranking produced by each methodology returns a similar result only for very few T-UCRs, like uc.8+, making necessary another step of scoring (section) for the definition of the best discriminating panel of T-UCRs. In Table 1 the first 24 T-UCRs are reported, using a cut-off of $50\%$ for the sum score of the T-UCRs expression with higher FC and of $60\%$ for the $\log _2(FC) = 1$, we identified 13 and 21 T-UCRs, respectively, to use in a panel for the prediction of BlCa in patients. In Additional file 1 the complete results by using $p\,value < 0.001$, $\log _2(FC) > 1.58$ and $\log _2(FC) > 1$ are described. We stress that the results of the ensembles models are obtained after applying fivefold cross-validation to the overall data labeled as BlCa (both LG and HG) and NBE. In Section 3 of Additional file 1 we detailed the parameters of the ensemble models.

**Table 1:**
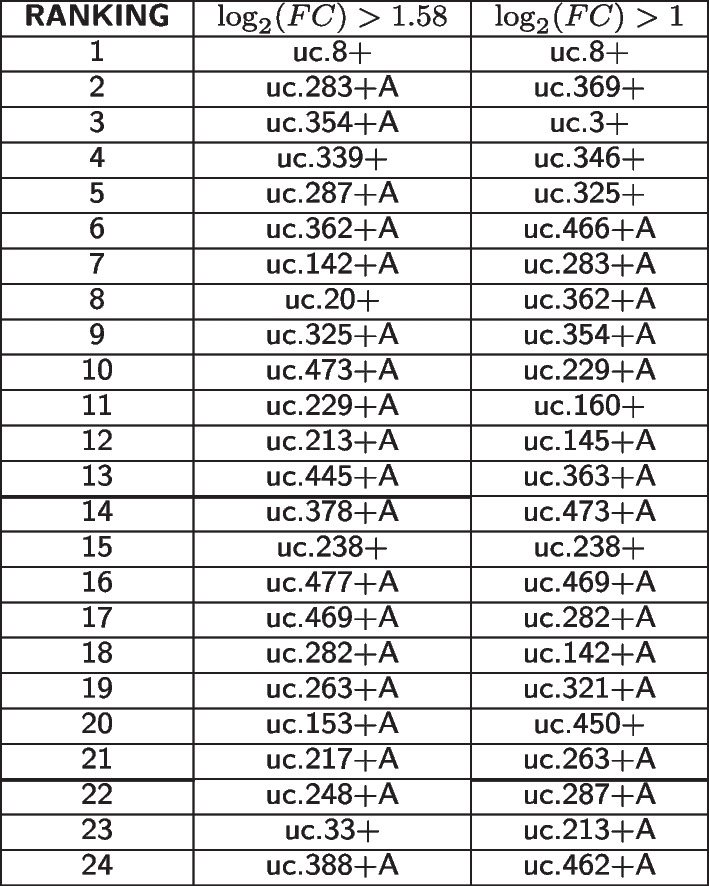
First 24 ranked T-UCRs by using different $log\_2(FC)$ values in the analysis with ensemble models and scoring

The T-UCRs up to bold lines in the two lists are the selected ones

## Discussion

**Fig. 3:**
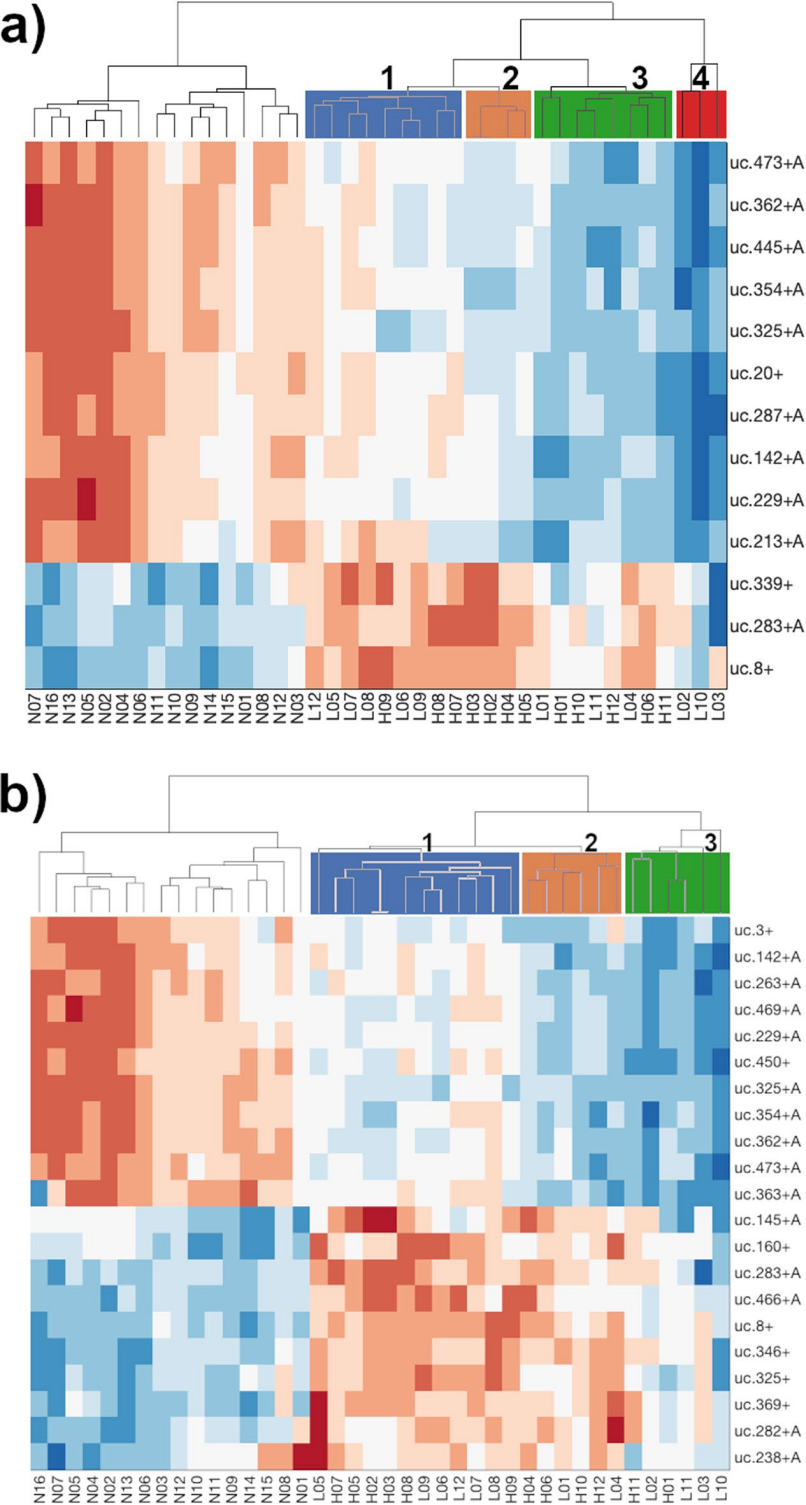
Heatmap plot of the T-UCRs expression in healthy people and BlCa patients. **a** Expression profile of the 13 T-UCRs selected by a *p* value cut-off $<0.001$, and **b** expression profile of the 21 T-UCRs selected by a *p* value cut-off $<0.01$. Dendrograms produced by hierarchical clustering identified groups of patients with similar T-UCRs expression. The groups are colored and numbered in accordance with the labeling of Fig. 1 panels a and b. N01 to N18 are the controls from healthy people, while L01 to L12 and H01 to H12 are the low and high grade BlCa patients, respectively. Red and blue colors represent high and low expression, respectively

Using the two panels of the newly identified T-UCRs, we reported in a Heatmap plot (Fig. 3a) their differential expression for each sample, where N01 to N18 are the control NBE samples, while L01 to L12 and H01 to H12 are the samples from Low and High Grade BlCa patients, respectively. Both panels clearly discriminate between the NBE and the BlCa (LG and HG) samples, returning two separate clusters. These Heatmaps are well characterized by the inversion of the T-UCRs expressions in the normal with respect to the cancerous bladder tissue. However, depending on the signature panel, the clustering of the different T-UCR expressions permitted also to identify four or three different groups of BlCa patients characterized by a specific expression of the T-UCRs in the signature panel. These clusters appear rather independent from the BlCa Grade associated with the patients, in fact, in the panel of 13 T-UCRs, excluding a single group with only LG patients, the other three include both BlCa Grade albeit in different ratios. In the panel comprising 21 T-UCRs,we identified only three heterogeneous groups including both LG and HG BlCa patients.

**Fig. 4:**
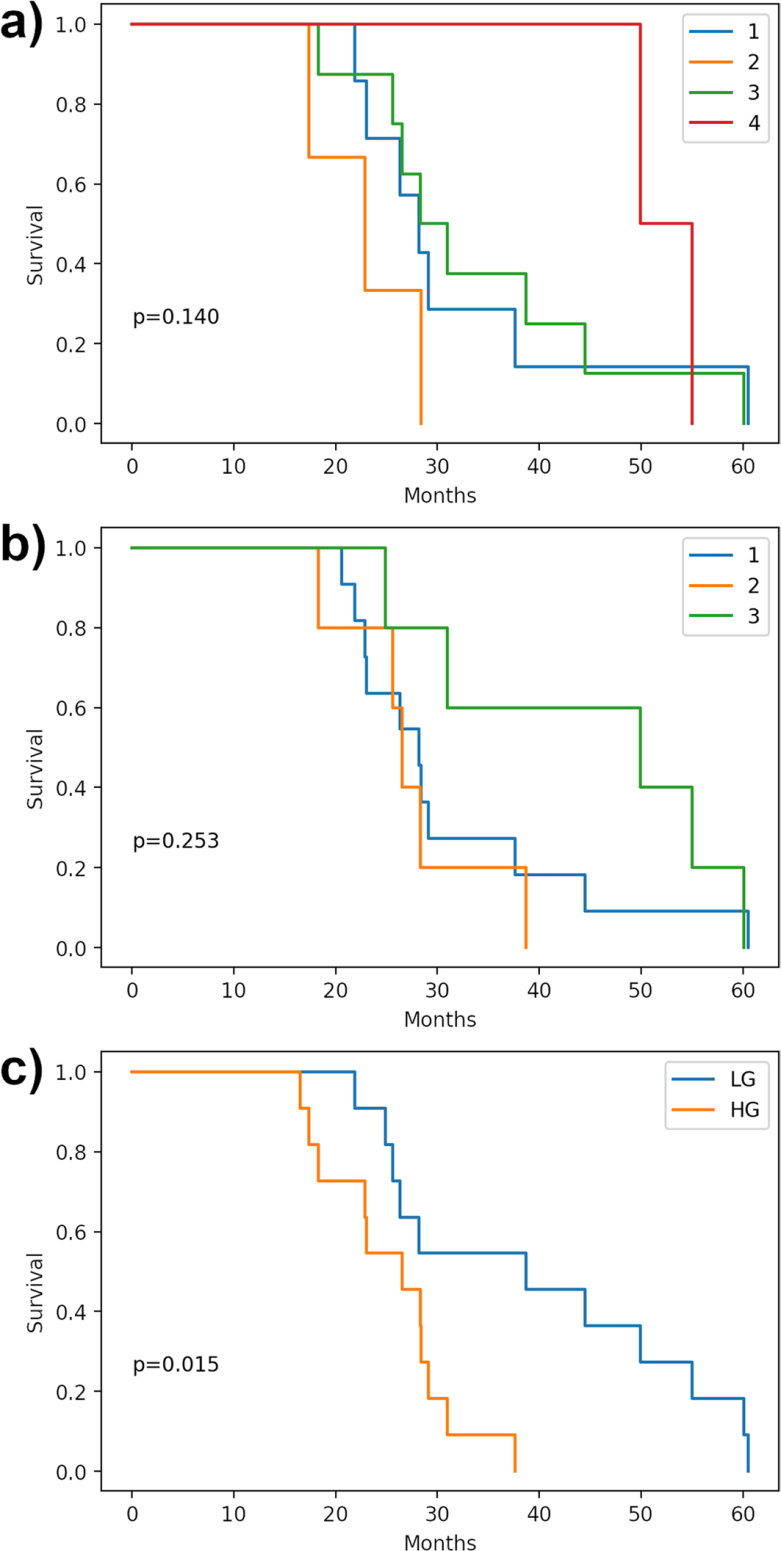
**a** Survival of BlCa patients groups as identified and indicated in Fig. 5a with the same colors and numbers. **b** Survival of BlCa patients groups as identified and indicated in Fig. 5b with the same colors and numbers. **c** Classical LG and HG BlCa patients survival distribution. The log-rank *p* values are indicated for each plot

**Fig. 5:**
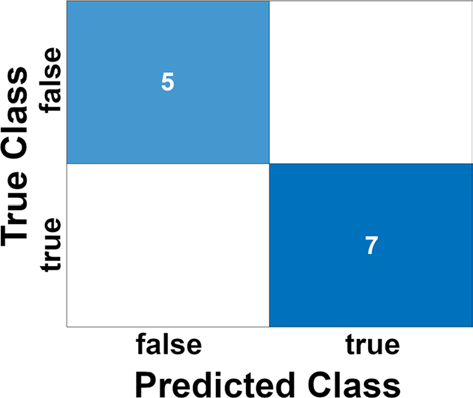
Confusion matrix: classification results by considering a logistic regression based model

In order to evaluate the potentiality of one or both panels as prognostic predictors of the BlCa progression in patients, we analyzed our results with respect to the relative patient survival. In Fig. 4a, the plot of patient survival with respect to the BlCa histological Grade distribution, was reported. Although it is possible to observe a difference relative to the diagnosed Grade of BlCa in the plot, this approach poorly discriminates between BlCa patients. In fact, although the trend is favorable for LG patients, half of them belonging to the LG group have a survival at about 30 months, which is the same as most of HG group patients. Thus, a similar result could make the communication about life expectancy useless. Differently, plotting the relative survival of the patients distributed according to the 4 groups identified by the 13 T-UCRs panel (Fig. 4b), we clearly identified a group of patients (number 2 in Fig. 4b) with very short lifespan ($<30$ months), and a group (number 4) with high lifespan ($>30$ months). The other two groups (numbers 1 and 3), even though showed a different T-UCRs expression, and are in the middle with a similar intermediate lifespan. Therefore, distributing the patients along the groups predicted by the T-UCRs panel, may open the possibility to obtain a more detailed evaluation of the disease progression regarding patient survival. Moreover, the identification of two groups (numbers 1 and 3) with similar survival, but with differences at molecular level, becomes interesting in the light of possible differentiated therapies. A less efficient rating between patients was obtained plotting the relative survival of patients according to the distribution in the 3 groups predicted by the 21 T-UCRs signature panel (Fig. 4c). This result suggested that despite the lower number of selected T-UCRs, the tighten parameters used for the T-UCRs selection, such as the higher FC ($\log _2(FC) > 1.58$) and the lower *p* value (0.001), has made the signature panel able to better discriminate between patients BlCa Grades. These observations are in agreement with the lower log-rank *p* values calculated for the groups of patients predicted by the 13 T-UCRs signature panel (Fig. 4). The use of more stringent thresholds also pays off in terms of efficiency, for the lower number of T-UCRs to be investigated and the reduced budget necessary for these analyses. As reported in Fig. 5, to confirm this hypothesis, the classification results obtained on a test set independently selected ($30\%$ of the overall data set) for each patient of the 13 selected T-UCRs are considered. We consider classification of bladder cancer (Low and High Grade) patient samples and normal bladder epithelium controls. The classification model is based on a logistic regression model. We achieve the $100\%$ of perfect classification highlighting as the selected T-UCRs permit to obtain reliable predictions (see Section 4 of Additional file 1).

## Conclusions

Implementation of Artificial Intelligence methodologies to the field of medical applications represents one of the most important challenges of our century. In this work we presented a methodology based on ML techniques for the selection of T-UCRs markers from microarray data. In the first phase T-UCRs, both statistically significant and significantly differentially expressed, are selected. In the second phase, statistical and ML based approaches (that is, logistic regression, Random Forest, XGBoost and LASSO) are used for ranking, by scoring methodology, to identify the relevant markers to be taken into account during the clinical practices. Among all the gene expressions obtained from the feature ranking, the uc.8+ stands out for its importance. This results aims for focusing on new automatic systems, which could support the prognosis and/or RNA-based BlCa therapy. Furthermore, our methodology could be applied to other forms of cancer which are characterized for abnormal expression of T-UCRs, such as in hematopoietic tissues (leukemia) [5], depending on the availability of data. However, considering that in normal human tissues, T-UCRs are expressed both ubiquitously and in a tissue-specific manner, different T-UCR panels are expected to characterize the various cancer forms. In the next future, the authors will perform this methodology on new patient-derived samples obtained by clinical practices and they will explore and compare new feature selection methodologies (e.g., sparse coding [17], learning with Bayesian framework [18]), and temporal behaviour of T-UCRs for robust prognosis [19]. Furthermore, the panel can be used for developing an eXplainable Artificial Intelligent (XAI) based decision support system that helps clinicians in diagnosis of bladder cancer from tissue or fluid samples also by user-friendly applications [20].

## Materials and methods

### Study population

In [4] the authors focused on the quantitative study of T-UCRs deregulated in tissue biopsies. The histological samples were obtained as described in [4, 6]. 24 BlCa patient samples, consisting of 12 Low Grade (LG) and 12 High Grade (HG), and 17 samples from normal bladder epithelium (NBE), were analyzed [4] (see Table 2 for the dataset summary). Patients’ data were classified using the TNM international classification system of malignant tumors, according to the classification criteria of the UICC (Union for International Cancer Control) providing the disease stage and grade.

**Table 2:** Dataset summary

Tissue samples analysed	41
BlCa patients	24
Low Grade	12
High Grade	12
Control (NBE)	17
Number of UCRs/sample	481
Transcripts/sample analysed	962
Total T-UCRs raw data analysed	39,442

### T-UCRs expression profiling

Trying to identify/build a diagnostic signature for BlCa early diagnosis, 962 sense and antisense transcripts of 481 known UCRs have been evaluated using a custom microarray (OSU-CCC 4.0), which included sense and antisense probes. The first one corresponding to the sense genomics sequence (named “+”) and the other one to the complementary sequence (named “+A”) for all the 481 human ultraconserved sequences reported by Bejerano et al. [3]. Each probe was spotted in duplicate in two different slide locations, and therefore quadruplicate numerical values were available for analysis [5]. Expressed T-UCRs were analysed using class comparison analysis with the BRB-ArrayTools software program (version 3.6.0). The criterion for inclusion of specific T-UCRs in the T-UCRs list was a *p* value less than 0.01. Normalized and raw T-UCRs data files were uploaded to the Gene Expression Omnibus (http://www.ncbi.nlm.nih.gov/geo) under GEO accession number GSE68594.

### Feature selection

**Fig. 6:**
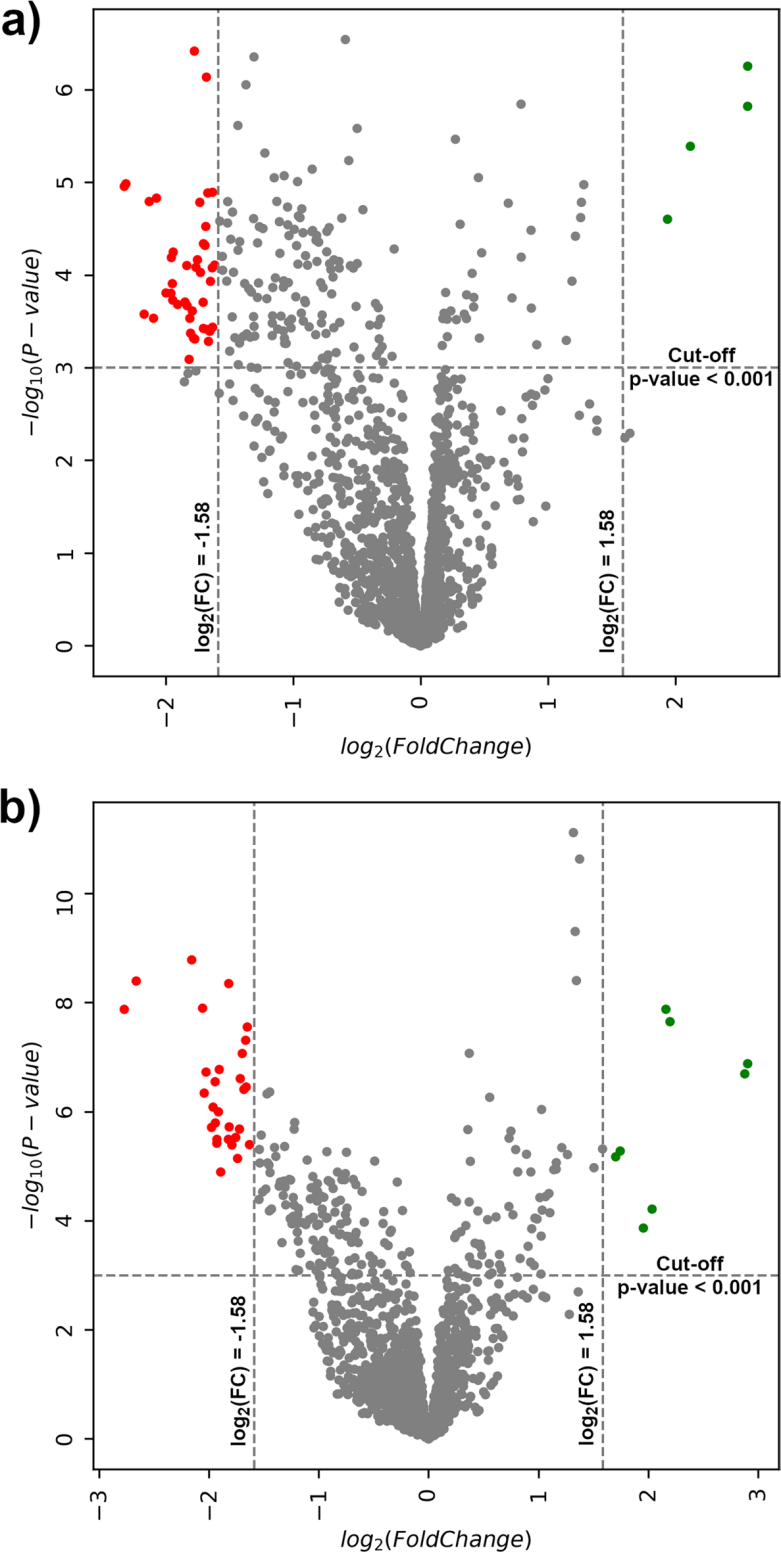
Volcano plots of the Fold Changes and *p* values of expressed T-UCRs in BlCa patients. **a** Gene expression for LG vs Normal, and **b** gene expression for HG vs Normal. The horizontal and vertical dashed lines represent the significance threshold (i.e. cut-off criteria). The differentially significant expressed T-UCRs are in green (high-expressed) and in red (low-expressed), while the insignificantly changed ones are in gray. Cut-off criteria: $-log_{10}(p\,value) > 3$, corresponding to a $p\,value < 0.001$, and $\log _2(FoldChange) > \pm 1.58$

A large amount of T-UCRs expression data from microarray experiments was analysed in order to obtain a differential expression profiling. Fold-Change (FC) and *t* test are the two main parameters for measuring differential expressions [21]. In particular, FC measures the quantity changes of gene expression comparing NBE and LG or HG BlCa patients. Genes are considered down-regulated if the expression ratio of a given gene in the two observed cases decreases, whereas genes are considered up-regulated, if the expression levels between two observed cases increases. An unpaired *t* test is applied for differential expression with a standard two-tailed test on every sequence in BlCa and NBE data obtaining a *p* value for each sequence. Using the FC and the *p* values, we determined the most differentially expressed T-UCRs, comparing the BlCa and the NBE samples. We adopt a cut-off criteria $-log_{10}(p\,value) > 3$, corresponding to a $p\,value < 0.001$, and $\log _2(FoldChange) > \pm 1.58$ (see Fig. 6 for an example). We use these restricted cut-off values in order to assign only a $\sim 30\%$ to the higher and lower T-UCRs expression area, respectively, increasing the selectivity of the selection.

### Feature relevance

Any ML method depends in part on the learning and generalization capacity of the implemented technique and in large part, on the characteristics of each observation. This definition is only partially true because providing a high number of features does not always lead to good learning. This is because the quality of the data, i.e., the expressive capacity of a feature against the data itself is often more important than the number of features. Including irrelevant elements increases the computational complexity and the problems of learning, therefore leading to problems of underfitting and overfitting. This type of behavior is recognizable in any type of task, whether it is a problem inherent in the data classification, data regression, or generation of new data. To deal with this kind of problem it is common to use feature importance techniques (also called feature relevance). Therefore feature importance aims to assign a score to input features of a model based on how significant they are at predicting a target variable [22] through data analysis techniques that can be based on statistical correlation scores [23, 24] or as happening in the last years ML are largely adapted [25]. In this work we consider an ensemble of feature importance models for reducing the number of T-UCRs selected in the previous step. The models are logistic regression, random forest, LASSO and XGBoost. Feature importance is used to improve the predictive model selecting those T-UCRs, pre-selected by the method in section, to delete (lowest scores) or those T-UCRs to keep (highest scores).

The feature importance models that we consider are:

- *Logistic regression* (LR) is a statistical model that in its basic form uses a logistic function to model the probability of a certain class or event [26]. Features importance are obtained by examining the coefficients of the model that are assigned to each input value. In particular, if an assigned coefficient is a large (negative or positive) number, it has some influence on the prediction, on the contrary, if the coefficient is zero, it does not have any impact on the prediction.
- *Least Absolute Shrinkage and Selection Operator* (LASSO) [27] consists of an approach to regression analysis that performs both variable relevance and regularization. In fact, LASSO regression contains a penalty term based on a $L_1$ norm (Manhattan distance) with the effect of actually forcing some of the coefficients to become zero, this is actually removing features from the model, and is effectively performing feature selection and relevance.
- *Random Forest* (RF) are an ensemble learning method for classification and regression operating by constructing a multitude of decision trees at training time [12]. Each tree of the random forest can calculate the importance of a feature according to its ability to increase the pureness of the leaves. Feature importance is calculated as the decrease in node impurity weighted by the probability of reaching that node. The node probability can be calculated by the number of samples that reach the node, divided by the total number of samples: the higher the value, the more important the feature.
- *eXtreme Gradient Boosting* (XGBoost) is the most efficient decision-tree-based ensemble ML algorithm that implements gradient boosting [28]. A benefit of using gradient boosting is that after the boosted trees are constructed, it is relatively straightforward to retrieve importance scores for each attribute. Importance is calculated for a single decision tree by the amount that each attribute split point improves the performance measure, weighted by the number of observations the node is responsible for.

## Supplementary Information

**Additional file 1.** A new biomarker panel of ultraconserved long non-coding RNA for bladder cancerprognosis by a machine learning based methodology, supplementary materials.

## Data Availability

For detailed information regarding the results, see the Additional file 1. A Python software package is available through GitHub at https://bit.ly/3Ny7mXe for the software, containing all the source codes used to run the ensemble of models.

## Abbreviations

lncRNA: Long non-coding RNA
T-UCR: Transcribed-Ultraconservative Region
BlCa: Bladder cancer
NBE: Normal bladder epithelium
LG: Low Grade
HG: High Grade
ML: Machine learning
LR: Logistic regression
LASSO: Least Absolute Shrinkage and Selection Operator
RF: Random Forest
XGBoost: EXtreme Gradient Boosting
XAI: EXplainable Artificial Intelligent
