# Supplementary material for: A new biomarker panel of ultraconserved long non-coding RNAs for bladder cancer prognosis by a machine learning based methodology
Source: BMC Bioinformatics. 2023 Mar 6;23(Suppl 6):569. doi: 10.1186/s12859-023-05167-6 (PMC9987036; doi:10.1186/s12859-023-05167-6)
Supplement: Supplementary file 1 — Additional file 1. A new biomarker panel of ultraconserved long non-coding RNA for bladder cancerprognosis by a machine learning based methodology, supplementary materials. [file 12859_2023_5167_MOESM1_ESM.pdf]

RESEARCH

A new biomarker panel of ultraconserved long non-coding RNA for bladder cancer prognosis by a machine learning based methodology. SUPPLEMENTARY MATERIALS

Angelo Ciaramella[1*], Emanuel Di Nardo[1,2], Daniela Terracciano[3], Lia Conte[4], Ferdinando Febbraio[5] and Amelia Cimmino[6]

Author details
[1]Department of Science and Technology, University of Naples "Parthenope", Centro Direzionale, Isola C4, 80143 Naples, Italy. [2]Department of Computer Science, University of Milan, Via Celoria, 18, 20133 Milan, Italy. [3]Department of Translational Medical Science, University of Naples "Federico II", Via Pansini 5, 80131 Naples, Italy. [4]Department of Experimental Urology - Radboud University Medical Center, Geert Grooteplein-Zuid 10, 6525GA Nijmegen, Netherlands. [5]Institute of Biochemistry and Cell Biology, CNR, Via Pietro Castellino 111, 80131 Naples, Italy. [6]Institute of Genetics and Biophysics, CNR, Via Pietro Castellino 111, 80131 Naples, Italy.

References

1. Nardone, D., Ciaramella, A., Staiano, A.: A sparse-modeling based approach for class specific feature selection. Peerj Computer Science **5:e237** (2019)
2. Bishop, C.M.: Pattern Recognition and Machine Learning. Springer, Cambridge CB3 0FB, U.K. (2006)
3. Breiman, L.: Random forests. Machine Learning **45**, 5–32 (2001)
4. Chen, T., C, G.: Xgboost: A scalable tree boosting system. In Proceedings of the 22nd ACM SIGKDD International Conference on Knowledge Discovery and Data Mining, 785–794 (2016)

*Correspondence: angelo.ciaramella@uniparthenope.it
[1]Department of Science and Technology, University of Naples "Parthenope", Centro Direzionale, Isola C4, 80143 Naples, Italy
Full list of author information is available at the end of the article

1 Features selection methods

In literature several features selection methods can be considered and some are [1]:

- **Logistic Regression** (LR)is a process of modeling the probability of a discrete outcome given an input variable [2].
- **LASSO** involves penalizing the absolute size of the regression coefficients and is usually used for creating parsimonious models in presence of a *large* number of features.
- **EN**: Elastic Net is a hybrid of ridge regression and LASSO regularization. Experimental studies have suggested that the Elastic Net technique can outperform LASSO on data with highly correlated features.
- **RFS**: Robust Feature Selection method is a sparse based-learning approach for feature selection which emphasizes the joint $\ell_{2,1}$ norm minimization on both loss and regularization function.
- **ls-$\boldsymbol{\ell_{2,1}}$**: ls-$\ell_{2,1}$ is a supervised sparse feature selection method. It exploits the $\ell_{2,1}$-norm regularized regression model for joint feature selection, from multiple tasks where the *classification objective function* is a quadratic loss.
- **ll-$\boldsymbol{\ell_{2,1}}$**: ll-$\ell_{2,1}$ is a supervised sparse feature selection method which uses the same concept of ls-$\ell_{2,1}$ but instead uses a *logistic loss* as *classification objective function*.
- **Fisher**: Fisher is one of the most widely used supervised filter feature selection methods. It selects each feature as the ratio of inter-class separation and intraclass variance, where features are evaluated independently and, the final feature selection occurs by aggregating the m top ranked ones.
- **Relief-F**: Relief-F is an iterative, randomized and supervised filter approach that estimates the quality of the features according to how well their values differentiate data samples that are near to each other; it does not discriminate among redundant features and performance decreases with few data.
- **mRmR**: Minimum-Redundancy-Maximum-Relevance is a mutual information filter based algorithm which selects features according to the maximal statistical dependency criterion.
- **MI**: Mutual Information is a non-negative value, which measures the dependency between the variables. Features are selected in a univariate way. The function relies on nonparametric methods based on entropy estimation from k-nearest neighbors distances.
- **SMBA-CSFS**: Sparse-Modeling Based Approach for Class Specific Feature Selection , that simultaneously exploits the idea of *Sparse Modeling* and *Class-Specific Feature Selection*.
- **Random Forest** used for classification and regression ant it is based on a multitude of decision trees [3].
- **eXtreme Gradient Boosting** (XGBoost) efficient decision-tree-based ensemble ML algorithm adopting gradient boosting [4].

2 Feature relevance and scoring

For improving the prognosis of the disease progression in BlCa patients, we need to rank the most significant T-UCRs identified by the volcano plot. Applying the ensemble of feature importance methodologies, we ranked the 70 and 24 T-UCRs

obtained varying the FC from 2 to 3, respectively. In Table 1 the first 70 T-UCRs are reported where $p - value < 0.001$ and $\log_2(FC) > 1$. In Table 2 we report the first 24 T-UCRs where $p - value < 0.001$, $\log_2(FC) > 1.58$. Refer to Figure 1 for an example of feature relevance by using LASSO on 24 T-UCRs selected with $p - value < 0.001$ and $\log_2(FC) > 1.58$.

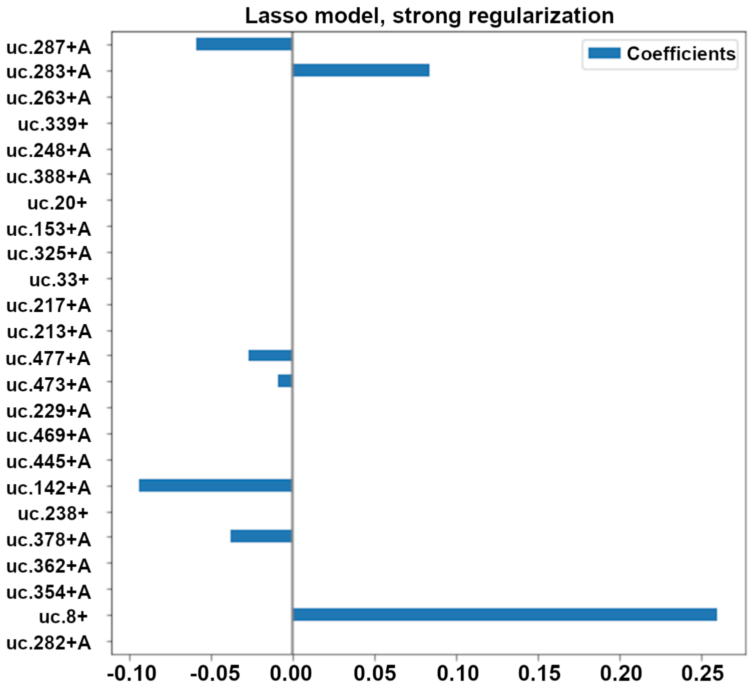

Figure 1: Example of feature relevance by using LASSO on 24 T-UCRs selected with $p - value < 0.001$ and $\log_2(FC) > 1.58$.

3 Ensemble models parameters

In the following the main parameters of the ensemble models are described.

3.1 *Logistic Regression*

- $l2$ penalty term;
- $dual\ formulation$ is False and it is adopted when $n_samples > n_features$;
- The maximum number of iterations $= 1000$;
- Tolerance for stopping criteria $= 1e - 4$;
- Inverse of regularization strength $= 1.0$;
- Constant (i.e., bias) added to the decision function;
- Optimization algorithm: Limited-memory BFGS (L-BFGS) of the family of quasi-Newton methods that approximates the Broyden–Fletcher–Goldfarb–Shanno algorithm (BFGS) using a limited amount of computer memory.

3.2 *LASSO*

- Constant that multiplies the L1 term $\alpha = 0.015$. $\alpha = 0$ is equivalent to an ordinary least square, solved by the Linear Regression object;

- Whether to calculate the intercept for this model is True;
- The maximum number of iterations = 1000;
- The tolerance for the optimization = $1e - 4$.

3.3 *Random Forest*

- The number of trees in the forest = 100;
- Mean squared error for quality of a split;
- Nodes are expanded until all leaves are pure or until all leaves contain less than the min samples split samples = 2;
- The minimum number of samples required to be at a leaf node = 1;
- The minimum weighted fraction of the sum total of weights (of all the input samples) required to be at a leaf node = 0;
- The number of features to consider when looking for the best split: $maxfeatures = numberoffeatures$;
- A node split if this split induces a decrease of the impurity greater than or equal to this value = 0.0;
- Whether bootstrap samples are used when building trees;
- Complexity parameter used for Minimal Cost-Complexity Pruning = 0.0.

3.4 *XGBoost*

- The initial prediction score of all instances, global bias = 0.5;
- Booster used: gbtree;
- Subsample ratio of columns for each level = 1;
- Subsample ratio of columns for each split = 1;
- Subsample ratio of columns when constructing each tree = 1;
- Boosting learning rate = 0.1;
- Maximum delta step we allow each tree's weight estimation to be = 0;
- Maximum tree depth for base learners = 3;
- Minimum sum of instance weight (hessian) needed in a child = 1;
- Value in the data which needs to be present as a missing value = $None$;
- Number of boosting rounds = 100;
- Number of parallel threads used to run xgboost = 1;
- Random number seed = 0;
- L1 regularization term on weights = 0;
- L2 regularization term on weights = 1;
- Balancing of positive and negative weight = 1;
- Subsample ratio of the training instance = 1;
- The degree of verbosity = 1.

4 ROC curve

Receiver operating characteristic (ROC) curves for logistic regression features selection for the the two used datasets (Fig. 2).

References

1. Nardone, D., Ciaramella, A., Staiano, A.: A sparse-modeling based approach for class specific feature selection. Peerj Computer Science **5:e237** (2019)
2. Bishop, C.M.: Pattern Recognition and Machine Learning. Springer, Cambridge CB3 0FB, U.K. (2006)
3. Breiman, L.: Random forests. Machine Learning **45**, 5–32 (2001)
4. Chen, T., C, G.: Xgboost: A scalable tree boosting system. In Proceedings of the 22nd ACM SIGKDD International Conference on Knowledge Discovery and Data Mining, 785–794 (2016)

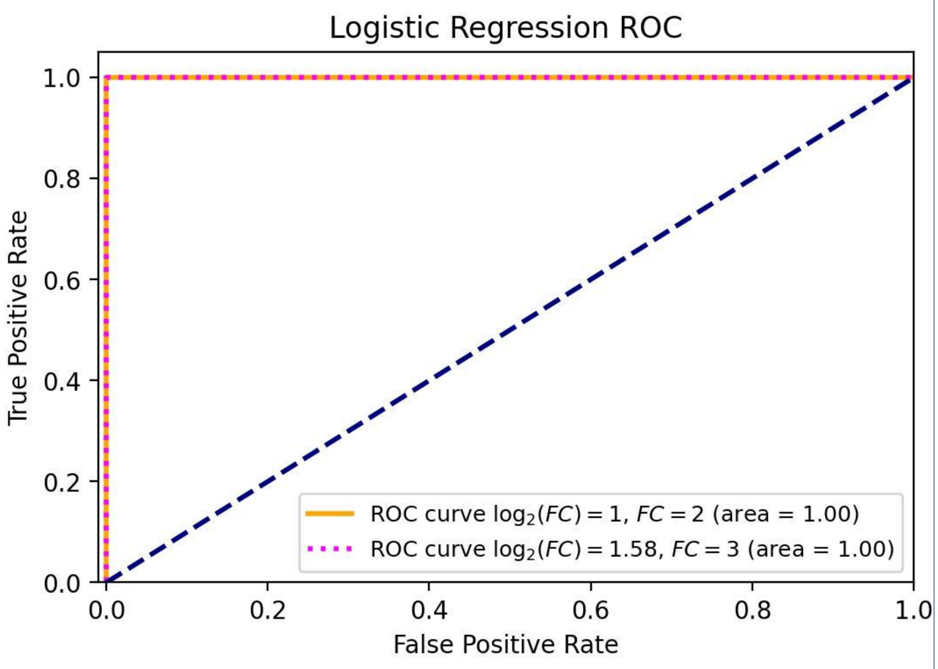

Figure 2: Receiver operating characteristic (ROC) curves for logistic regression features selection for the the two used datasets. According to the classification scores, the obtained results allow to demonstrate a high sensitivity with a maximum area under the curve (AUC).

RANKING	textbfLASSO	LOGISTIC REGRESSION	RANDOM FOREST	XGBoost
1	uc.160+	uc.8+	uc.325+A	uc.346+
2	uc.3+	uc.283+A	uc.466+A	uc.354+A
3	uc.8+	uc.160+	uc.346+	uc.369+
4	uc.142+A	uc.145+A	uc.369+	uc.3+
5	uc.145+A	uc.339+	uc.354+A	uc.8+
6	uc.363+A	uc.369+	uc.8+	uc.325+A
7	uc.369+	uc.325+	uc.283+A	uc.362+A
8	uc.372+	uc.466+A	uc.153+A	uc.298+
9	uc.473+A	uc.3+	uc.217+A	uc.392+A
10	uc.283+A	uc.263+A	uc.145+A	uc.268+
11	uc.200+	uc.346+	uc.362+A	uc.195+
12	uc.469+A	uc.229+A	uc.160+	uc.238+
13	uc.325+	uc.282+A	uc.287+A	uc.450+
14	uc.190+A	uc.213+A	uc.363+A	uc.466+A
15	uc.229+A	uc.142+A	uc.177+A	uc.404+
16	uc.238+	uc.450+	uc.33+	uc.213+A
17	uc.189+	uc.230+	uc.263+A	uc.163+
18	uc.234+	uc.287+A	uc.325+	uc.275+A
19	uc.378+A	uc.363+A	uc.462+A	uc.325+
20	uc.362+A	uc.288+A	uc.229+A	uc.469+A
21	uc.445+A	uc.20+	uc.404+	uc.445+A
22	uc.43+	uc.217+A	uc.282+A	uc.190+A
23	uc.354+A	uc.238+	uc.106+A	uc.189+
24	uc.477+A	uc.473+A	uc.3+	uc.142+A
25	uc.106+A	uc.195+	uc.445+A	uc.229+A
26	uc.230+	uc.362+A	uc.20+	uc.43+
27	uc.374+A	uc.462+A	uc.473+A	uc.234+
28	uc.4+	uc.469+A	uc.16+	uc.378+A
29	uc.282+A	uc.31+	uc.392+A	uc.473+A
30	uc.21+A	uc.378+A	uc.48+A	uc.230+
31	uc.138+	uc.248+A	uc.457+	uc.374+A
32	uc.206+A	uc.477+A	uc.89+	uc.4+
33	uc.346+	uc.33+	uc.268+	uc.282+A
34	uc.342+	uc.153+A	uc.4+	uc.21+A
35	uc.298+	uc.342+	uc.388+A	uc.138+
36	uc.466+A	uc.325+A	uc.469+A	uc.206+A
37	uc.462+A	uc.448+A	uc.138+	uc.342+
38	uc.450+	uc.392+A	uc.477+A	uc.462+A
39	uc.28+	uc.354+A	uc.288+A	uc.106+A
40	uc.213+A	uc.374+A	uc.468+	uc.28+
41	uc.48+A	uc.163+	uc.298+	uc.477+A
42	uc.287+A	uc.48+A	uc.238+	uc.217+A
43	uc.263+A	uc.177+A	uc.163+	uc.363+A
44	uc.163+	uc.34+A	uc.450+	uc.287+A
45	uc.275+A	uc.298+	uc.374+A	uc.283+A
46	uc.339+	uc.106+A	uc.31+	uc.263+A
47	uc.404+	uc.200+	uc.372+	uc.339+
48	uc.96+	uc.96+	uc.96+	uc.96+
49	uc.268+	uc.190+A	uc.213+A	uc.200+
50	uc.468+	uc.372+	uc.206+A	uc.468+
51	uc.31+	uc.206+A	uc.21+A	uc.31+
52	uc.288+A	uc.43+	uc.248+A	uc.288+A
53	uc.456+A	uc.468+	uc.200+	uc.456+A
54	uc.448+A	uc.138+	uc.34+A	uc.448+A
55	uc.44+A	uc.44+A	uc.189+	uc.48+A
56	uc.217+A	uc.412+A	uc.412+A	uc.44+A
57	uc.248+A	uc.275+A	uc.28+	uc.160+
58	uc.412+A	uc.456+A	uc.190+A	uc.248+A
59	uc.392+A	uc.404+	uc.378+A	uc.372+
60	uc.388+A	uc.189+	uc.339+	uc.412+A
61	uc.457+	uc.268+	uc.44+A	uc.388+A
62	uc.89+	uc.28+	uc.234+	uc.145+A
63	uc.20+	uc.4+	uc.342+	uc.457+
64	uc.16+	uc.457+	uc.142+A	uc.89+
65	uc.177+A	uc.388+A	uc.456+A	uc.20+
66	uc.153+A	uc.21+A	uc.230+	uc.16+
67	uc.325+A	uc.234+	uc.195+	uc.177+A
68	uc.33+	uc.445+A	uc.448+A	uc.153+A
69	uc.195+	uc.89+	uc.275+A	uc.33+
70	uc.34+A	uc.16+	uc.43+	uc.34+A

Table 1: First 70 ranked T-UCRs by using $p - value < 0.001$ and $\log_2(FC) > 1$ values in the analysis with ensemble models (Lasso, Logistic Regression, Random Forest and XGBoost)

RANKING	textbfLASSO	LOGISTIC REGRESSION	RANDOM FOREST	XGBoost
1	uc.8+	uc.8+	uc.8+	uc.354+A
2	uc.142+A	uc.283+A	uc.325+A	uc.8+
3	uc.283+A	uc.339+	uc.354+A	uc.325+A
4	uc.287+A	uc.282+A	'uc.283+A	uc.283+A
5	uc.378+A	uc.263+A	uc.153+A	uc.238+
6	uc.477+A	uc.229+A	uc.445+A	uc.362+A
7	uc.473+A	uc.142+A	uc.388+A	uc.213+A
8	uc.20+	uc.213+A	uc.33+	uc.339+
9	uc.445+A	uc.287+A	uc.282+A	uc.20+
10	uc.263+A	uc.20+	uc.229+A	uc.287+A
11	uc.354+A	uc.469+A	uc.362+A	uc.469+A
12	uc.362+A	uc.238+	uc.473+A	uc.378+A
13	uc.339+	uc.473+A	uc.339+	uc.142+A
14	uc.238+	uc.362+A	uc.217+A	uc.445+A
15	uc.248+A	uc.217+A	uc.378+A	uc.477+A
16	uc.469+A	uc.248+A	uc.248+A	uc.229+A
17	uc.153+A	uc.477+A	uc.213+A	uc.473+A
18	uc.229+A	uc.325+A	uc.20+	uc.217+A
19	uc.388+A	uc.354+A	uc.477+A	uc.33+
20	uc.213+A	uc.33+	uc.287+A	uc.153+A
21	uc.217+A	uc.378+A	uc.469+A	uc.388+A
22	uc.33+	uc.153+A	uc.142+A	uc.248+A
23	uc.325+A	uc.445+A	uc.238+	uc.263+A
24	uc.282+A	uc.388+A	uc.263+A	uc.282+A

Table 2: First 24 ranked T-UCRs by using $p - value < 0.001$ and $\log_2(FC) > 1.58$ values in the analysis with ensemble models (Lasso, Logistic Regression, Random Forest and XGBoost)
